# The Mission of the Psychologist

**Published:** 1857-10-01

**Authors:** Forbes Winslow


### THE JOURNAL

OF

### PSYCHOLOGICAL MEDICINE

AND

### MENTAL PATHOLOGY.

OCTOBER 1, 1857.

### ART. I.—THE MISSION OF THE PSYCHOLOGIST:

*Being the Address delivered by* DR. FORBES WINSLOW, *on his taking the chair as President of the Association of Medical Officers of Asylums and Hospitals for the Insane, at the Annual Meeting, held in London, July 2nd,* 1857.

GENTLEMEN,—I have the honour of occupying on this occasion the distinguished position of your President, and in that capacity it is now my privilege and pleasure to appear before you.

Addressing myself to a body of gentlemen distinguished for their ability, experience, and knowledge of the morbid phenomena of mind, as well as practical acquaintance with the treatment of the insane, I ask, is it possible for me to give you any information you are not already fully in possession of; is it in my power to impress upon your mind a higher appreciation of the noble and honourable vocation in which we are all engaged than that which I believe you have already formed? I despair of bringing before this association any novel facts in pathology or therapeutics—any startling deductions calculated to excite your interest, attract your attention, or instruct your understandings. However, I will, notwithstanding the obvious disadvantages under which I labour,—with, I trust, an unostentatious distrust in my own capacity either to inform or please—venture to address to my fellow-labourers in the great work of love and Christian philanthropy a few words in relation to the anxious, onerous, and often painful duties which devolve upon all engaged in the treatment of the insane. It is well that we should, from time to time, whilst occupied in life's pilgrimage, lean upon our staff, pause, and seriously consider the position it has pleased the will of Providence that we should occupy. It is right and befitting that we should occasionally solemnly reflect upon the PAST, dwell with earnestness upon the PRESENT, and seriously ponder over the FUTURE. In commercial phraseology, it is right that we should occasionally take stock, examine carefully our ledger, ascertain with accuracy the balance at the banker's, and consider with business-like precision and exactitude our credit and debtor account. The process of mental retrospection cannot be otherwise than beneficial to us all. It is well that the man occupied in the higher spheres of usefulness, who is cultivating the more abstruse and philosophical departments of the science and art of medicine, that he who is entrusted by the legislature with the care and treatment of the insane, should frequently ask himself the questions—What are the functions delegated to me? Do I entertain a right appreciation of my important duties, and am I so discharging them that at the great and final day of judgment I shall be in a position to give a good and faithful account of my stewardship?

Considering our vocation in its strictly scientific relations, need I observe, when comparing it with other branches of our noble profession of which it forms a part, that the practical psychologist occupies high and honourable vantage ground.

It is not my intention to breathe a word in disparagement of other sections of the medical profession. Each class holds an honourable rank in the great circle of science; each division has allotted to it its own anxious and specific duties; and whatever position the practitioner of medicine may fill, whether it be that of a surgeon, a general or special physician, all in their separate and respective spheres of duty have responsible functions devolving upon them. But in what respect do we differ from other departments of the medical profession? what particular and specific functions are assigned to those engaged in the treatment of the insane? Whilst the physician devoted to general practice is administering to the *physical* state of the system—in healing abnormal conditions of *matter* interfering with the *vital* manifestations—we, as psychologists, take a more exalted flight into the regions of science. It is our enviable privilege to deal with the *human mind*—to study its healthy as well as disordered state, to investigate that SPIRITUAL aura, that DIVINE ESSENCE which is so mysteriously interwoven and associated with the grosser particles of the material fabric.

How noble is the study in which we are engaged! how important the duties that devolve upon us! how solemnly responsible is our position! Is it possible to exaggerate or over-estimate our character, influence, importance, and dignity! What profound and accurate knowledge of the mind in its normal state do we not require before we are fitted successfully to investigate, unravel, and treat remedially its deviations from a healthy standard! How intimate must be our acquaintance with the phenomena of thought, and with the nature and operations of the passions! How exact should be our notions of the instinctive and perceptive faculties before we are fully qualified to appreciate subtle, morbid, psychical conditions!

We should entertain right notions of our duty and position; we should encourage elevated, lofty thoughts and grand conceptions of our honourable vocation; we should impress repeatedly, earnestly, and emphatically upon our own understandings and the minds of all engaged in the same holy work the significant fact, that we are occupied in the study and treatment of a class of diseases affecting the very source, spring, and fountain of that principle which in its healthy operations alone can bring us into remote proximity to DEITY—that we have to deal with the spiritual part of man's complex nature, with that which elevates him in the scale of created excellences, and places him high on the pedestal among the great, the good, and the wise. But our solemn functions expand in interest, gravity, and importance, as we reflect that it is mind prostrated, perverted, and often crushed by disease with which we, as practical physicians, have to deal; that we have placed under our care a class of the afflicted human family, reduced by the inscrutable decrees of Providence to the most humiliating, degrading, and helpless position to which poor human nature can fall; that it is our duty to witness the sad wreck of great and noble minds, and the decay of exalted genius. Like the historian and antiquarian wandering with a sad heart over ground made classical and memorable in the story of great men, and in the annals of heroic deeds—surveying with painful interest the crumbling ruins of ancient temples—viewing with subdued emotion the almost extinguished remains of proud imperial cities, consecrated by the genius of men renowned in the world's history as scholars, artists, philosophers, and poets, so it is our duty to wander through the sad ruins of still greater temples than any that were in ancient days raised to the honour of an unseen DEITY. Yes, it is our distressing province to witness great and good intellects, and proud understandings, levelled to the earth and crumbled like dust in the balance, under the dire influence of disease. Survey that old man crouched in the corner, with his face buried in his hands. He is indifferent to all that is passing around him—he heeds not the voice of man nor of woman—he delights not in the carolling of birds nor in the sweet music of the rippling brooks. The gentle wind of heaven, playing its sweetest melody as it rushes through the greenwood, awakens no consciousness of nature's charms. Approach and speak to him. Address him in terms of endearment and affection—bring before him the glowing images of the past. He elevates his head, gazes listlessly and mechanically at you,

"makes no sign," and, dropping his poor head, buries it in his bosom, and sinks into his former moody state of melancholy abstraction. This man's oratory charmed the senate—the magic of his eloquence held thousands in a state of breathless admiration; his influence was commanding, his sagacity and judgment eminently acute and profound. View him as he is fallen from his high and honourable estate. Listen to the sweet and gentle voice of yonder woman, upon whose head scarcely eighteen summer suns have shed their genial warmth and influence. How merrily she dances over the greensward! How touchingly she warbles, like poor *Ophelia*, sweet snatches of song! What a pitiful spectacle of a sweet mind lying in fragments before us! Look, she has decked herself with a spring garland. Now she holds herself perfectly erect, and walks with queenly majesty. Approach her side, accost her, she exclaims, "Yes, he will come; he promised to be here; where are the guests? where's the ring? where's my wedding dress—my orange flowers?" Suddenly her mind is over-shadowed, and her face assumes an expression of deep choking and bitter anguish—she alternately sobs and laughs—is gay, sad, cheerful, and melancholy—

> "Thought and affliction, passion, hell itself,
> She turns to favour and to prettiness."

Speak again to her, and another change takes place in the spirit of her dream. Like her sad prototype, the sweetest creation of Shakspeare's immortal genius, she plaintively sings—

> "He is dead and gone, lady,
> He is dead and gone;
> At his head a green grass turf,
> At his heels a stone."

Her history is soon told. Deep and absorbing passion, elevated hopes, bright and fanciful dreams of the future—DEATH with all its sad trappings and solemn mockery—seared affections, a broken heart, and a disordered brain! In its sad ruin her mind retains much of its native purity, innocence, and sweetness.

It is not my object to bring before you painful, fanciful, and imaginative sketches.

The two illustrations I have cited are faithful and truthful outlines of cases that must have come under the notice of us all. How keenly cases like these tear the heartstrings asunder and call into active operation all the kindly sympathies of our nature.

Having considered thus briefly the character of our vocation and the grave responsibilities of our position, I would with great submission to the members of this association dwell shortly on the present state of that section of psychological science more immediately connected with the practical pursuits in which we are in common engaged—viz., that of the care and treatment of the insane.

At the onset I would premise that, as a body of men engaged in a holy and sacred office, we must not close our eyes to the fact that our position is not what we have a right to expect or are entitled to claim. Our studies, beyond a doubt, are ennobling and elevating—our duties, if conscientiously discharged, excite into action the tenderest feelings of the *heart*, and the highest capacities of the *intellect.* To an intimate knowledge of the general characteristics of disease, and the sciences of pathology and therapeutics, which we possess in common with other sections of our profession, the psychological physician must unite a profound knowledge, not only of the mind, but of mind as manifesting itself in *character* and human nature, in the most enlarged acceptation of these terms. He has to battle with the intellect in a condition of aberration; he has to combat with passions in a state of morbid exaltation; he has to administer to the feelings, affections, and appetites in a deranged or perverted condition. He has, in the exhibition of his moral remedial agents, emphatically to act upon *mind* as well as upon *matter;* and if he be unqualified by natural aptitude, education, habits of thought, and careful study of the higher branches of philosophy, to perform such duties, he is obviously unfit for the post he is called upon to occupy. If such are the recognised characteristics of the psychological physician, why is he considered by the public, to a certain extent, as a man engaged in the pursuits of commerce and trade? How is it that a psychological expert, when in the witness-box, is so often snubbed and browbeaten? Why should we, when engaged in the practical execution of our duties, be viewed and estimated as persons pursuing a degrading and dishonourable calling? Why should the finger of derision and scorn be pointed at us? Why should we be singled out from the crowd, and have flung in our faces the odious, offensive, and repulsive designation of "mad doctor," when called upon as experts to assist in the solemn administration of justice? I ask, why such a state of things should exist? why men engaged in so honourable, sacred, and dignified a pursuit should occasionally find themselves in a position so false, painful, and humiliating? In justice to ourselves, as well as to those unhappy persons confided to our care, we are bound to consider this matter with becoming seriousness. The question cannot be ignored. There must be something "rotten in the state" to justify such a sad condition of things. We do not occupy our legitimate position in public estimation, and it is our duty to ask why such should be the case? Having given this question much anxious consideration and thought, I have come to the following conclusions:—According to my apprehension, there are THREE modes of accounting for our present status. In the first place, I attribute much of the existing evil to the conduct of a few narrow-minded and ignorant men, who have improperly had the care of the insane, and who have by their very questionable proceedings in a measure degraded us all to their own ignoble level. Have we been true to ourselves? Is it necessary that we should look much away from home to find the adverse causes that have been operating to our degradation and disparagement? Have we not made merchandize of the insane, considering their care and treatment more as a question of commerce than of science? Gentlemen, I am occasionally overpowered with feelings of deep humiliation and shame, when I take up the advertisement sheet of the daily newspapers, and see to what measures men will resort to bring themselves, their houses, and their asylums prominently before the public, with a view to their personal aggrandizement. Not satisfied with advertising their establishments in the glowing, fanciful, poetical, and flowery language of the auctioneer, they go a step in advance, and offer liberal per centages and bonuses to all medical men patronizing their institutions. Again, how often we see asylums and their unhappy inmates brought into the market and offered for sale like a flock of sheep to the highest bidder, in a manner calculated to destroy all public confidence and trust in the honesty, integrity, and even common respectability of those connected with similar institutions. Consider for a moment the practical effect upon the *public* mind, and by reflex action upon the position of the psychological physician, of the following advertisement, which has been the round of the medical journals:

"INSANITY.—Twenty per cent. annually on the receipts will be guaranteed to any Medical Man recommending a quiet Patient, of either sex, to a First-Class Asylum, with the highest testimonials. Address — — ——."

This is not an isolated illustration. No number of the *Times* appears without containing announcements of a similar character. Thank God! the great body of men engaged in the treatment of the insane would sooner permit themselves to be reduced to the lowest depths of poverty and distress than resort to such unprofessional means to advance their pecuniary interests in life.

If we desire to elevate ourselves in the estimation of good men, if it be our object to secure for our specialty a legitimate position in public opinion, it behoves us to enter our firm protest against disgraceful proceedings like these; to hold no converse, companionship, or communion with men who thus degrade themselves to the condition of the common trader and shopkeeper, without any portion of the respectability, honesty, and worth which so commonly distinguish men engaged in the legitimate pursuits of commerce.

To remedy this great and growing evil we must in the first place put our own houses in order—

> "Our remedies oft in ourselves do lie
> Which we ascribe to Heaven; the fated sky
> Gives us free scope; only doth backward pull
> Our slow designs, when we ourselves are dull."

It is now my duty to consider the second cause operating to our disadvantage—viz., the effect of legislative enactments upon the character of the psychologists and the condition of the insane.

The legislature has never fully recognised or admitted the important principle that insanity is a *pathological* condition; in other words, that it is a type of diseased manifestation. This great first principle should be prominently recorded in the preamble of every parliamentary enactment relating to the treatment of the insane, and all legislation should be based upon the full and liberal recognition of the fact that *insanity, lunacy, unsoundness of mind, idiotcy, imbecility*—to use the common legal phraseology—are curable states of bodily and brain disease, disordering the manifestations of the mind; and that in the organization of all institutions for the care and treatment of the insane, as well as in the distribution of licences to persons willing to undertake the management of this class of affections, the first question to be considered is, whether the party is fitted by education, knowledge, and experience for the performance of his responsible duties. I would permit no one to have the legal charge and treatment of either an acute or chronic case of mental aberration who was not a qualified medical man. As long as licences are granted to non-professional persons, as well as to women, the public will be indisposed to believe that insanity is the result of a physical morbid condition of the brain, or of some organ in close sympathy with it; or that the disease is one amenable to remedial medical treatment. The non-recognition of this important elementary principle in the past legislation on this subject of lunacy has undoubtedly had the effect, not only of encouraging in the public mind erroneous views of the nature and treatment of insanity, but of placing the psychological physician in a false commercial position. And why should such be the case? The qualified and educated medical practitioner who has an asylum for the treatment of his own patients finds himself placed in the same category with non-professional men and women, into whose hands are entrusted the legal custody and, treatment of the insane. It is obvious that this course of procedure must inevitably tend to depreciate the character of all connected with asylums, lower the psychologist in public estimation, and tend to discountenance all remedial treatment.

What has been the natural consequence of permitting non-professional persons to have the care of the insane? Persons palpably unfitted for the right and humane performance of so solemn a trust have been discovered seriously and wilfully neglecting the interest of those entrusted to their legal guardianship. The evil has been fully recognised by the State, and from time to time various legislative enactments for the protection of the insane have become part of the statute law of the land, so constructed as to meet the exigencies of the case, and, if possible, avert a recurrence of these evils. Stringent legal clauses have found their way into these various lunacy enactments, until we may be said to act under the authority of a bill of *pains and penalties*. I do not complain of the operation of these measures; I refer to the fact simply with a view of establishing my position, that owing to the character of a few of those who have in former years had the care and treatment of the insane, such stringent laws have been deemed essentially necessary for their safety and protection.

It is not my intention to consider in detail the various existing lunacy bills for the purpose of satisfying you that the provisions of the present law operate prejudicially to the interests of psychology, and are seriously detrimental to those connected with the care of the insane. I will cite but one illustration of the fact.

Agreeably to the provisions of a former enactment, no medical man was held to be legally qualified for the post of a Commissioner in Lunacy who had any interest, direct or indirect, in the confinement of the insane for *one* year previously. This clause was altered in the last Act of Parliament, the one now in operation; and in conformity with the amended bill, no medical man is statutorily eligible for the office of Commissioner in Lunacy who has had for two years an interest, direct or indirect, in the confinement of the insane; in other words, the candidate must have been disconnected with a private asylum for a period of *two* years, the legislature not considering one year a sufficient time to restore the mind of the psychological physician to a state of judicial purity!

So great is the contamination and degradation incidental to a connexion with the treatment of the insane and the management of an asylum, that the legislature in its profound wisdom and extraordinary sagacity, considers two years' purgation—two years of psychological *quarantine* necessary before the medical man can be viewed as qualified to present a *clean bill of health,* and thereby fitted to sit at the Board of Commissioners, and assist in the administration of the law! Upon what principle was such a clause introduced into the Lunacy Bill?

I fully admit that no person appointed to so important an office should have the most remote interest, direct or indirect, in the care and treatment of the insane, and that before accepting an appointment of the kind, and prior to his taking the oaths of office,he should be in a position to say that he has entirely ceased to have the slightest or faintest shadow of interest in the confinement of any one insane individual; but it puzzles my simple understanding to comprehend why the law should require *two* years of cleansing and purification on the part of gentlemen engaged in the solemn and faithful discharge of the highest class of professional duties before they can be considered fitted for such an appointment.

I should be insulting the understanding of those I have the honour to address if I were to occupy any time in attempting to demonstrate the practically injurious effect of such a provision of the law upon the character and position of all engaged in the study of psychology, and in the care and treatment of the insane. Whatever tends to lower in public estimation the psychological physician, whether connected or unconnected with a private asylum, must materially, and without doubt, injuriously affect also those connected with our public institutions, and at the same time damage seriously the vital interests of the insane. Apart from the mischief such a state of the law must inflict upon the great body of psychological physicians, consider for one moment the serious injustice it does to a number of physicians engaged in private practice, and who, in a measure, are compelled to be interested in and associated with private asylums. These men are disfranchised, virtually excluded from the office of commissioner. Irrespectively of a man's reputation, character, and experience, he is legally disqualified if he retains any interest in the confinement of a single insane person. Destroy by legislative enactments the *social* position of the physician engaged in this branch of practice, and you immediately cripple his resources, and very much circumscribe his sphere of usefulness.

I have no hesitation in asserting that this is an unjust, a mischievous, and an iniquitous enactment. I can conceive a man of European reputation, of great practical knowledge, of unbounded experience, of profound sagacity, of high and unimpeachable honour and character, looking forward at the close of a brilliant and useful career to an appointment of this nature, as one of the prizes which should be awarded to professional men whose great public services and talents were entitled to some slight recognition. This man would be ineligible for the office the duties of which he was admirably fitted to discharge, unless for two years he had ceased to have any interest in the confinement of the insane! Profound legislators! Wise statesmen! Eminent and sagacious senators! to have conceived so enlightened and benevolent an enactment.

In considering the third cause which has operated to the disadvantage of the psychologist I must be brief. The ignorance exhibited by the public of the real characteristics of insanity and of the treatment necessary for its cure is certainly great. Poets, dramatists, and novelists have materially aided in promulgating fictitious, imaginative, and consequently erroneous notions of insanity.

From this imputation I, of course, except our own immortal Shakspeare, that great magician whose colossal genius, profound wisdom and subtlety,—whose playful fancy, brilliant wit, extraordinary and intimate insight into the secret workings of the human mind and heart, and whose universal knowledge, shed a brilliant flood of light upon every subject to which he directed the powers of his noble and transcendant intellect. His delineations of insanity must ever be viewed as master creations—as imperishable monuments of grandeur, purity, beauty, grace, loveliness, and truth. He was pre-eminently the great and gifted psychologist of his epoch, and no man (and we have had great giants since his day) has yet been able distantly to approach him in his knowledge of healthy or morbid mental phenomena.

In conclusion, I would again repeat that we must look faithfully at our own hearts, honestly analyse our own motives, and conscientiously scrutinise our own conduct, if we desire to discover the true cause of the present unsatisfactory status of the psychological physician, and are anxious to elevate our body in the social scale.

Having said so much about ourselves, let me finally add a few words respecting those sad cases placed under our special care and protection. We cannot too frequently allow our minds to dwell upon the peculiar state of those reduced by insanity to a condition of utter and childish helplessness. In other classes of disease, in which the functions of the brain remain intact, the invalid, even while suffering the most acute and agonizing pain, bodily distress, and physical prostration, is in a state to appreciate his actual relations with those around him—he feels sensitively the exhibition of tender sympathy—he properly estimates the care and attention bestowed upon his case, and recognises the skill of his faithful medical adviser. Alas! how different are the feelings and thoughts of many of the insane! In this class of affections the kindness, sympathy, skill, unremitting assiduity, and attention of the physician are often not outwardly or manifestly appreciated. He has, in many cases, to pursue his holy work without the exhibition of the slightest apparent consciousness, on the part of the patient, of his efforts to assuage his anguish and mitigate his condition of mental disease and bodily suffering. Nevertheless, it is our sacred duty, even where, as is occasionally the case, our actions are greatly misconstrued and perverted by those to whose relief we are administering, to unflaggingly persevere in our efforts to carry out our curative process of treatment. Our poor, unhappy invalid may believe that we are acting the part of his bitterest foe. This ought not to excite in our mind any feeling but that of the most profound love and sympathy. If his language be offensive and repulsive —if he be guilty of any acts of violence towards those in attendance upon him—let us never for a moment lose sight of the fact, that his unhappy affliction has, to a degree, destroyed his free will, and that he, for a time, has ceased to be a responsible being. It would be cruel, whilst such a condition of mind exists, to treat such a patient otherwise than as a person deprived by disease of the power of complete self-government and moral control. I feel how unnecessary it is for me to urge upon those connected with this association, as well as to all engaged in the treatment of the insane, the importance of never losing sight of the fact, that even in the worst form of mental disease there are some salient and bright spots upon which we may act, and against which we may direct our most potent curative agents. How true it is that

> "There is some soul of goodness in things evil,
> Would men observingly distil it out."

The more formidable, and apparently hopelessly incurable types of mental derangement admit, if not of cure, at least of considerable alleviation and mitigation. It is always in our power to materially add to the physical comforts of even the worst class of patients; and when a cure is impracticable, it is our duty by every means in our power to ease the passage to the tomb. Again, we should never say of a case of insanity that it is incurable, or that it baffles our skill. We undoubtedly possess the power of materially modifying (if we cannot entirely re-establish the mental equilibrium) the most unfavourable and distressing forms of insanity—rendering the violent, and turbulent, tractable and amenable to discipline—the dangerous, harmless—the noisy, quiet—the dirty, cleanly in their habits, and the melancholy, cheerful. It is possible by a careful study of the bodily and mental idiosyncrasy of each individual case, and by an unremitting attention to dietetic and hygienic regimen, as well as by a persevering, unflagging, and assiduous administration of physical and moral remedies for their relief, to

> "Pluck from the memory a rooted sorrow;
> Raze out the written troubles of the brain,
> And with some sweet oblivious antidote
> Cleanse the foul bosom of that perilous stuff
> Which weighs upon the heart."

The spirit of love, tender sympathy, Christian benevolence, unwearying kindness, and warm affection, should influence our every thought, look, and action, when engaged in the treatment of the sad and distressing cases entrusted to our care. We should never forget that it is the special province of the psychological physician to

> "*Fetter strong madness in a silken thread,*
> *Charm ache with air, and agony with words.*"

Oh! what a holy, honourable, and sacred occupation is that in which we all have the privilege to be engaged! The angels in heaven might well envy us the ennobling and exalted pleasures incidental to our mission of love and charity.

---

